# Bis[μ-2-(aminosulfanyl)pyridine(1−)]bis­[(η^5^-penta­methyl­cyclo­penta­dien­yl)iridium(III)] diiodide

**DOI:** 10.1107/S1600536809037167

**Published:** 2009-09-19

**Authors:** Yusuke Sekioka, Takayoshi Suzuki

**Affiliations:** a Department of Chemistry, Graduate School of Science, Osaka University, Toyonaka 560-0043, Japan; b Division of Chemistry and Biochemistry, Graduate School of Natural Science and Technology, Okayama University, Okayama 700-8530, Japan

## Abstract

In the title dinuclear iridium(III) complex, [Ir_2_(C_10_H_15_)_2_(C_5_H_5_N_2_S)_2_]I_2_, the iridium(III) atoms are bridged by 2-(aminosulfanyl)pyridine(1−) [(2-py)SNH] ligands in a μ-(2-py)SNH-κ^2^ *N*(py),*N*(NH):κ*N*(NH) mode. The dinuclear complex cation lies on a crystallographic inversion center, resulting in a planar Ir_2_N_2_ ring with an Ir—N(py) bond length of 2.085 (9) Å and bridging Ir—N(NH) bonds of 2.110 (9) and 2.113 (9) Å. The two (2-py)S units have mutually *anti* configurations with respect to the Ir_2_N_2_ ring

## Related literature

For nitro­gen-atom transfer, see: Du Bois *et al.* (1997 ▶); Birk & Bendix (2003 ▶). For photolysis of iridium(III) azido complexes, see: Kotera *et al.* (2008 ▶); Sekioka *et al.* (2005 ▶); Suzuki *et al.* (2003 ▶). For related organic compounds, see: Robinson & Hurley (1965 ▶); Brito *et al.* (2002 ▶); Miura *et al.* (2003 ▶). For related coordination compounds, see: Nakayama *et al.* (1999 ▶); Esquivias *et al.* (2007 ▶); Nanthakumar *et al.* (1999 ▶); Ishiwata *et al.* (2006 ▶); Arita *et al.* (2008 ▶). For 2-pyridylmethyl­amido complexes showing the μ-κ^2^ *N*(py),*N*(NH):κ*N*(NH) bridging mode, see: Westerhausen *et al.* (2002 ▶); Wong & Wong(2002 ▶).
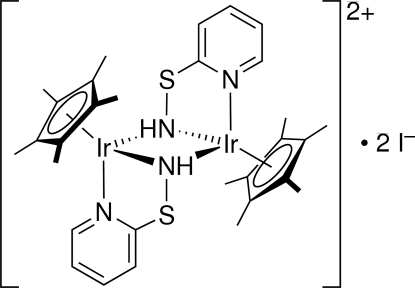


## Experimental

### 

#### Crystal data

[Ir_2_(C_10_H_15_)_2_(C_5_H_5_N_2_S)_2_]I_2_
*M* *_r_* = 1158.98
Monoclinic, 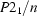

*a* = 12.839 (3) Å
*b* = 12.169 (3) Å
*c* = 11.299 (4) Å
β = 102.754 (19)°
*V* = 1721.8 (8) Å^3^
*Z* = 2
Mo *K*α radiation
μ = 9.66 mm^−1^
*T* = 296 K
0.20 × 0.10 × 0.08 mm

#### Data collection

Rigaku AFC7R diffractometer
Absorption correction: ψ scan (North *et al.*, 1968 ▶) *T*_min_ = 0.248, *T*_max_ = 0.512
5294 measured reflections
5006 independent reflections
3621 reflections with *I* > 2σ(*I*)
*R*_int_ = 0.080
3 standard reflections every 150 reflections intensity decay: none

#### Refinement

*R*[*F* ^2^ > 2σ(*F* ^2^)] = 0.062
*wR*(*F* ^2^) = 0.212
*S* = 1.02
5006 reflections
182 parameters
H-atom parameters constrained
Δρ_max_ = 3.47 e Å^−3^
Δρ_min_ = −3.49 e Å^−3^

### 

Data collection: *WinAFC Diffractometer Control Software* (Rigaku, 1999 ▶); cell refinement: *WinAFC Diffractometer Control Software*; data reduction: *CrystalStructure* (Rigaku/MSC, 2004 ▶); program(s) used to solve structure: *SIR92* (Altomare *et al.*, 1994 ▶); program(s) used to refine structure: *SHELXL97* (Sheldrick, 2008 ▶); molecular graphics: *ORTEP-3* (Farrugia, 1997 ▶); software used to prepare material for publication: *SHELXL97*.

## Supplementary Material

Crystal structure: contains datablocks global, I. DOI: 10.1107/S1600536809037167/zs2007sup1.cif

Structure factors: contains datablocks I. DOI: 10.1107/S1600536809037167/zs2007Isup2.hkl

Additional supplementary materials:  crystallographic information; 3D view; checkCIF report

## Figures and Tables

**Table 1:** Selected bond angles (°)

N1—Ir1—N2^i^	84.3 (3)
N1—Ir1—N2	77.6 (3)
N2^i^—Ir1—N2	74.1 (4)
Ir1^i^—N2—Ir1	105.9 (4)

Symmetry code: (i) 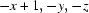.

**Fig. 1.**
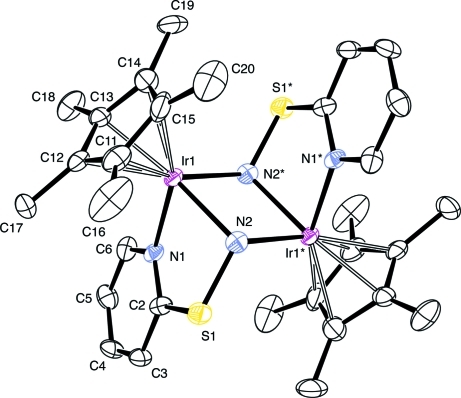
An ORTEP-3 (Farrugia, 1997) view of the cationic part of the compound (I), showing the atom numbering scheme. H atoms are omitted for clarity. Displacement ellipsoids are drawn at the 30% probability level. The asterisk (*) corresponds to symmetry code (-x + 1, -y, -z).

## supplementary crystallographic information

### Comment

Nitrogen-atom transfer is one of the promising synthetic methodologies for nitrogen-containing organic/inorganic compounds (Du Bois *et al.*, 1997; Birk & Bendix, 2003). To this end we are trying to prepare high-valent iridium nitrido (or nitrenido) complexes and are investigating their reactivities at the N atom site (Suzuki *et al.*, 2003; Sekioka *et al.*, 2005; Kotera *et al.*, 2008). In our previous paper (Sekioka *et al.*, 2005) it was reported that photolysis of an acetonitrile solution of [Cp*Ir(2-Spy)(N_3_)] (2-Spy = 2-pyridinethiolate) resulted in insertion of an N atom derived from the azido ligand into the Ir–N(py) bond to afford [Cp*Ir(1-N-2-Spy)]. The reaction solution containing this complex was mixed with P(OMe)_3_ and MeI in this order and several yellow crystals of compound (I) were deposited from the mixture, although the yield was very low (*ca* 2%). The single-crystal X-ray analysis revealed that (I) is an iodide salt of a dinuclear iridium(III) complex bridged by 2-pyridylthioamide(1–), [(Cp*Ir)_2_{µ-(2-py)SNH}_2_]I_2_, as shown in Fig. 1. The H atom of the bridging amide ligand (–NH) could not be located in the difference Fourier map. However, the observation of a ν(NH) band at 3073 cm^-1^ in the IR spectrum and the good agreement of elemental analysis with the calculated values for the diiodide salt, it is suggested that the bridging N atom is protonated to form the amide(1-) ligand. The mechanism for formation of (2-py)SNH^-^ is unknown at present but it could be a by-product of photolysis of the [Cp*Ir(2-Spy)(N_3_)] complex, or a N atom (or a NH-group) transfer product from the reaction of the [Cp*Ir(1-N-2-Spy)] complex with P(OMe)_3_ and MeI.

In compound (I), the N–*S* and *S*–C bond lengths of the bridging 2-pyridylthioamido(1–) ligand are 1.747 (9) and 1.73 (1) Å, respectively. 2-Pyridylthioamine [alternatively, 2-pyridinesulfenamide: (2-py)SNH_2_] was prepared by oxidation of 2-pyridinethiolate by chloramine, and its cobalt(II) and iron(II) complexes as well as their Schiff base derivatives {(2-py)SN=C*R*^1^*R*^2^} were also synthesized more than 40 years ago (Robinson & Hurley, 1965). However, none of the compounds containing (2-py)SNH_2_ have been so far characterized by X-ray analysis. The only example of the X-ray structural determination of a 2-pyridinesulfenamide is the *N*-piperidine derivative, (5-NO_2_-2-py)SNC_5_H_10_ (Brito *et al.*, 2002) in which the N–*S* and *S*–C bond lengths are 1.699 and 1.761 Å, respectively. The crystal structures of the related aminyl radicals, (2-py)SN(C_6_H_2_Ph_2_R), have also been reported (Miura *et al.*, 2003) in which the N–*S* and *S*–C bond lengths are 1.599 (4)–1.626 (8) and 1.770 (6)–1.781 (10) Å, respectively. Thus, in (I) the N–S bond is relatively longer, while the S–C bond is slightly shorter than those in similar organic compounds.

In the related transition-metal complex containing the 2-pyridylthioamide(1–) derivative, a cobalt(III) complex with tridentate (2-pyS)_2_N^-^ ligands, [Co{(2-pyS)_2_N}_2_]ClO_4_, has been structurally determined (Nakayama *et al.*, 1999). In this complex the N–*S* and *S*–C bond lengths are 1.711 (3)–1.718 (3) and 1.742 (4)–1.747 (4) Å, respectively. Furthermore, a few crystal structures of metal complexes with 2-pyridinesulfonamide {(2-py)SO_2_NH_2_} derivatives have been reported (Esquivias *et al.*, 2007; Nanthakumar *et al.*, 1999), but compound (I) is the first example containing coordinated (2-py)SNH^-^ ligand has been characterized by X-ray methods. The Ir–N(py) bond length in compound (I) is 2.085 (9) Å, and the bridging Ir–N(NH) bond lengths are 2.110 (9) and 2.113 (9) Å. As seen in Fig. 1, the (2-py)SNH^-^ ligand adopts a µ-κ^2^*N*(py),*N*(NH):κ*N*(NH) bridging mode. This coordination mode is rare, to our knowledge having precedent in only two 2-pyridylmethylamido (2-pyCHRNH^-^) complexes (Westerhausen *et al.*, 2002; Wong *et al.*, 2002). For the dinuclear iridium(III) complexes bridged by two amide-N donors, Ishikawa, Arita and coworkers reported the sulfonamido-bridged complexes (Ishiwata *et al.* 2006; Arita *et al.*, 2008). In their *p*-MeC_6_H_3_SO_2_NH-bridged dinuclear complex, [(Cp*Ir)_2_(µ-MeC_6_H_3_SO_2_NH)_2_], the two Cp* ligands are in mutually *syn* orientations with respect to the Ir_2_N_2_ ring, but in (I) the two Cp* ligands adopt an *anti* configuration .

### Experimental

A solution of [Cp*Ir(N_3_)(2-Spy)] (59 mg, 0.12 mmol) in dry acetonitrile (2 cm^3^) was prepared under a nitrogen atmosphere and photolyzed for 15 h with a high pressure Hg lamp (Riko UVL-100HA) at a temperature below 0 °C, controlled by a Yamato Neocool model BD12. To the resulting dark red solution was added P(OMe)_3_ (35 µ*L*, 0.30 mmol), the color of the mixture immediately turning to yellowish brown. After allowing the solution to stand at ambient temperature for 18 h, methyl iodide (18.5 µ*L*, 0.30 mmol) was added, and then the mixture was allowed to stand overnight. Several yellow crystals of [(Cp*Ir)_2_(2-pySNH)_2_]I_2_ (I) were deposited from the mixture. Yield: 1.5 mg (2.1%). Anal. Found: C, 31.17; H, 3.54; N, 5.08%. Calcd for C_30_H_40_I_2_Ir_2_N_4_S_2_: C, 31.09; H, 3.48; N, 4.83%. IR (Nujol): ν(NH) = 3073 cm^-1^.

### Refinement

The H atoms were located geometrically and constrained to ride on their parent atoms with N–H = 0.91 Å and C–H = 0.93–0.96 Å with *U*_iso_(H) = 1.2 *U*_eq_(N or C). The largest peak and deepest hole in the difference Fourier map (3.47 and -3.49 eÅ^-3^) are located 0.82 and 0.82 Å respectively, from atom Ir1.

### Crystal data

**Table:**

[Ir_2_(C_10_H_15_)_2_(C_5_H_5_N_2_S)_2_]I_2_	*F*(000) = 1080
*M**_r_* = 1158.98	*D*_x_ = 2.235 Mg m^−^^3^
Monoclinic, *P*2_1_/*n*	Mo *K*α radiation, λ = 0.71069 Å
Hall symbol: -P 2yn	Cell parameters from 25 reflections
*a* = 12.839 (3) Å	θ = 15.1–17.0°
*b* = 12.169 (3) Å	µ = 9.66 mm^−^^1^
*c* = 11.299 (4) Å	*T* = 296 K
β = 102.754 (19)°	Plate, yellow
*V* = 1721.8 (8) Å^3^	0.20 × 0.10 × 0.08 mm
*Z* = 2	

### Data collection

**Table:**

Rigaku AFC7R diffractometer	3621 reflections with *I* > 2σ(*I*)
Radiation source: rotating anode	*R*_int_ = 0.080
graphite	θ_max_ = 30.0°, θ_min_ = 2.7°
ω–2θ scans	*h* = −17→18
Absorption correction: ψ scan (North *et al.*, 1968)	*k* = 0→17
*T*_min_ = 0.248, *T*_max_ = 0.512	*l* = −15→6
5294 measured reflections	3 standard reflections every 150 reflections
5006 independent reflections	intensity decay: none

### Refinement

**Table:**

Refinement on *F*^2^	Primary atom site location: structure-invariant direct methods
Least-squares matrix: full	Secondary atom site location: difference Fourier map
*R*[*F*^2^ > 2σ(*F*^2^)] = 0.062	Hydrogen site location: inferred from neighbouring sites
*wR*(*F*^2^) = 0.212	H-atom parameters constrained
*S* = 1.02	*w* = 1/[σ^2^(*F*_o_^2^) + (0.1625*P*)^2^] where *P* = (*F*_o_^2^ + 2*F*_c_^2^)/3
5006 reflections	(Δ/σ)_max_ = 0.001
182 parameters	Δρ_max_ = 3.47 e Å^−^^3^
0 restraints	Δρ_min_ = −3.49 e Å^−^^3^

### Fractional atomic coordinates and isotropic or equivalent isotropic displacement parameters (Å^2^)

**Table:**

	*x*	*y*	*z*	*U*_iso_*/*U*_eq_	
Ir1	0.44162 (3)	0.04859 (3)	0.10915 (3)	0.03051 (16)	
I1	0.43226 (8)	−0.33173 (7)	0.19002 (9)	0.0577 (3)	
S1	0.6607 (2)	0.1519 (2)	0.0834 (3)	0.0444 (6)	
N1	0.5900 (6)	−0.0006 (7)	0.2111 (8)	0.0347 (17)	
N2	0.5455 (7)	0.0923 (7)	−0.0044 (8)	0.0360 (17)	
C2	0.6748 (8)	0.0563 (8)	0.1992 (11)	0.037 (2)	
C3	0.7773 (9)	0.0412 (11)	0.2784 (13)	0.053 (3)	
C4	0.7850 (9)	−0.0313 (12)	0.3700 (13)	0.054 (3)	
C5	0.7009 (11)	−0.0948 (10)	0.3812 (11)	0.049 (3)	
C6	0.6016 (10)	−0.0797 (10)	0.3026 (10)	0.046 (2)	
H2	0.5119	0.1449	−0.0563	0.043*	
H3	0.8367	0.0801	0.2670	0.064*	
H4	0.8493	−0.0379	0.4267	0.064*	
H5	0.7093	−0.1484	0.4412	0.059*	
H6	0.5435	−0.1222	0.3110	0.055*	
C11	0.3852 (11)	0.2107 (12)	0.1475 (14)	0.058 (3)	
C12	0.4010 (9)	0.1434 (11)	0.2566 (11)	0.046 (3)	
C13	0.3359 (9)	0.0464 (9)	0.2330 (12)	0.044 (3)	
C14	0.2687 (12)	0.0600 (12)	0.1115 (14)	0.061 (4)	
C15	0.3039 (13)	0.1527 (13)	0.0590 (12)	0.062 (4)	
C16	0.4361 (18)	0.3130 (13)	0.133 (2)	0.103 (9)	
C17	0.4780 (12)	0.1724 (16)	0.3763 (15)	0.082 (6)	
C18	0.3265 (16)	−0.0390 (11)	0.3278 (17)	0.067 (4)	
C19	0.1763 (11)	−0.016 (2)	0.059 (2)	0.092 (7)	
C20	0.2544 (17)	0.2022 (18)	−0.0683 (16)	0.102 (8)	
H16A	0.4104	0.3393	0.0513	0.123*	
H16B	0.5119	0.3024	0.1474	0.123*	
H16C	0.4199	0.3658	0.1890	0.123*	
H17A	0.5124	0.2411	0.3677	0.098*	
H17B	0.5309	0.1157	0.3970	0.098*	
H17C	0.4390	0.1787	0.4394	0.098*	
H18A	0.2779	−0.0956	0.2912	0.080*	
H18B	0.3002	−0.0048	0.3920	0.080*	
H18C	0.3954	−0.0705	0.3602	0.080*	
H19A	0.1715	−0.0727	0.1177	0.111*	
H19B	0.1881	−0.0498	−0.0135	0.111*	
H19C	0.1110	0.0247	0.0413	0.111*	
H20A	0.2943	0.2659	−0.0816	0.122*	
H20B	0.1815	0.2226	−0.0718	0.122*	
H20C	0.2568	0.1484	−0.1298	0.122*	

### Atomic displacement parameters (Å^2^)

**Table:**

	*U*^11^	*U*^22^	*U*^33^	*U*^12^	*U*^13^	*U*^23^
Ir1	0.0272 (2)	0.0357 (2)	0.0303 (2)	0.00425 (13)	0.00992 (14)	0.00336 (13)
I1	0.0620 (5)	0.0496 (5)	0.0624 (6)	−0.0088 (4)	0.0159 (4)	0.0109 (4)
S1	0.0439 (14)	0.0407 (13)	0.0502 (15)	−0.0105 (11)	0.0143 (11)	−0.0015 (11)
N1	0.031 (4)	0.033 (4)	0.043 (5)	0.005 (3)	0.015 (3)	−0.003 (3)
N2	0.036 (4)	0.032 (4)	0.040 (4)	0.007 (3)	0.008 (3)	0.006 (3)
C2	0.030 (5)	0.035 (5)	0.049 (6)	−0.001 (3)	0.012 (4)	0.000 (4)
C3	0.025 (5)	0.074 (9)	0.059 (8)	−0.002 (5)	0.007 (5)	−0.023 (6)
C4	0.027 (5)	0.076 (9)	0.051 (7)	0.002 (5)	−0.007 (5)	−0.010 (6)
C5	0.058 (7)	0.044 (6)	0.040 (6)	0.006 (5)	0.000 (5)	0.001 (5)
C6	0.054 (7)	0.046 (6)	0.039 (5)	0.012 (5)	0.014 (5)	0.004 (5)
C11	0.051 (7)	0.059 (8)	0.067 (8)	0.016 (6)	0.024 (6)	0.012 (6)
C12	0.034 (5)	0.056 (6)	0.053 (6)	0.004 (5)	0.020 (5)	−0.012 (5)
C13	0.032 (5)	0.048 (6)	0.052 (7)	0.002 (4)	0.011 (5)	−0.011 (5)
C14	0.047 (7)	0.081 (10)	0.055 (8)	0.009 (6)	0.007 (6)	−0.018 (7)
C15	0.068 (9)	0.081 (10)	0.045 (7)	0.015 (7)	0.028 (6)	−0.008 (7)
C16	0.14 (2)	0.045 (8)	0.16 (2)	0.032 (10)	0.095 (18)	0.038 (10)
C17	0.048 (8)	0.128 (16)	0.066 (9)	−0.007 (8)	0.007 (7)	−0.059 (10)
C18	0.086 (12)	0.045 (7)	0.083 (11)	−0.004 (7)	0.052 (10)	0.012 (7)
C19	0.030 (6)	0.138 (17)	0.112 (16)	−0.018 (9)	0.021 (8)	−0.055 (14)
C20	0.104 (15)	0.132 (17)	0.070 (11)	0.086 (14)	0.022 (10)	0.045 (11)

### Geometric parameters (Å, °)

**Table:**

Ir1—N1	2.085 (9)	C14—C15	1.40 (2)
Ir1—N2	2.113 (9)	C14—C19	1.52 (2)
Ir1—N2^i^	2.110 (9)	C15—C20	1.56 (2)
Ir1—C11	2.177 (14)	N2—H2	0.9100
Ir1—C12	2.182 (11)	C3—H3	0.9300
Ir1—C13	2.154 (13)	C4—H4	0.9300
Ir1—C14	2.231 (15)	C5—H5	0.9300
Ir1—C15	2.147 (15)	C6—H6	0.9300
S1—C2	1.730 (11)	C16—H16A	0.9600
S1—N2	1.747 (9)	C16—H16B	0.9600
N1—C2	1.322 (13)	C16—H16C	0.9600
N1—C6	1.396 (15)	C17—H17A	0.9600
C2—C3	1.431 (16)	C17—H17B	0.9600
C3—C4	1.35 (2)	C17—H17C	0.9600
C4—C5	1.357 (19)	C18—H18A	0.9600
C5—C6	1.395 (17)	C18—H18B	0.9600
C11—C12	1.457 (19)	C18—H18C	0.9600
C11—C15	1.46 (2)	C19—H19A	0.9600
C11—C16	1.43 (2)	C19—H19B	0.9600
C12—C13	1.437 (16)	C19—H19C	0.9600
C12—C17	1.531 (18)	C20—H20A	0.9600
C13—C14	1.46 (2)	C20—H20B	0.9600
C13—C18	1.515 (18)	C20—H20C	0.9600
			
N1—Ir1—N2^i^	84.3 (3)	C12—C13—Ir1	71.7 (7)
N1—Ir1—N2	77.6 (3)	C14—C13—Ir1	73.4 (8)
N2^i^—Ir1—N2	74.1 (4)	C18—C13—Ir1	128.9 (9)
N1—Ir1—C11	117.0 (5)	C12—C13—C14	106.3 (12)
N2—Ir1—C11	100.1 (4)	C12—C13—C18	124.4 (13)
N2^i^—Ir1—C11	156.7 (5)	C14—C13—C18	128.2 (13)
N1—Ir1—C12	94.2 (4)	C13—C14—Ir1	67.7 (7)
N2—Ir1—C12	128.3 (4)	C15—C14—Ir1	68.2 (9)
N2^i^—Ir1—C12	156.8 (4)	C19—C14—Ir1	130.6 (10)
N1—Ir1—C13	105.5 (4)	C13—C14—C15	108.2 (13)
N2—Ir1—C13	166.1 (4)	C13—C14—C19	122.9 (16)
N2^i^—Ir1—C13	119.5 (4)	C15—C14—C19	129.0 (17)
N1—Ir1—C14	143.3 (5)	C11—C15—Ir1	71.4 (8)
N2—Ir1—C14	139.0 (5)	C14—C15—Ir1	74.7 (9)
N2^i^—Ir1—C14	105.0 (4)	C20—C15—Ir1	128.0 (9)
N1—Ir1—C15	156.1 (5)	C11—C15—C14	110.5 (13)
N2—Ir1—C15	106.5 (4)	C11—C15—C20	122.0 (17)
N2^i^—Ir1—C15	119.6 (5)	C14—C15—C20	126.8 (17)
C11—Ir1—C12	39.1 (5)	C2—C3—H3	121.1
C11—Ir1—C13	66.2 (5)	C4—C3—H3	121.1
C11—Ir1—C14	64.3 (5)	C3—C4—H4	119.3
C11—Ir1—C15	39.4 (6)	C5—C4—H4	119.3
C12—Ir1—C13	38.7 (4)	C4—C5—H5	120.0
C12—Ir1—C14	63.4 (5)	C6—C5—H5	120.0
C12—Ir1—C15	64.4 (5)	C5—C6—H6	120.1
C13—Ir1—C14	38.9 (5)	N1—C6—H6	120.1
C13—Ir1—C15	65.1 (5)	C11—C16—H16A	109.5
C14—Ir1—C15	37.1 (6)	C11—C16—H16B	109.5
C2—S1—N2	94.9 (5)	C11—C16—H16C	109.5
C2—N1—C6	118.7 (10)	H16A—C16—H16B	109.5
C2—N1—Ir1	117.8 (7)	H16A—C16—H16C	109.5
C6—N1—Ir1	122.8 (7)	H16B—C16—H16C	109.5
Ir1^i^—N2—Ir1	105.9 (4)	C12—C17—H17A	109.5
S1—N2—Ir1	109.2 (4)	C12—C17—H17B	109.5
S1—N2—Ir1^i^	119.6 (4)	C12—C17—H17C	109.5
S1—N2—H2	107.2	H17A—C17—H17B	109.5
Ir1^i^—N2—H2	107.2	H17A—C17—H17C	109.5
Ir1—N2—H2	107.2	H17B—C17—H17C	109.5
N1—C2—S1	118.6 (8)	C13—C18—H18A	109.5
C3—C2—S1	119.2 (9)	C13—C18—H18B	109.5
N1—C2—C3	122.2 (11)	C13—C18—H18C	109.5
C2—C3—C4	117.8 (11)	H18A—C18—H18B	109.5
C3—C4—C5	121.4 (11)	H18A—C18—H18C	109.5
C4—C5—C6	119.9 (12)	H18B—C18—H18C	109.5
C5—C6—N1	119.7 (12)	C14—C19—H19A	109.5
C12—C11—Ir1	70.7 (7)	C14—C19—H19B	109.5
C15—C11—Ir1	69.2 (8)	C14—C19—H19C	109.5
C16—C11—Ir1	125.7 (11)	H19A—C19—H19B	109.5
C12—C11—C15	104.6 (13)	H19A—C19—H19C	109.5
C12—C11—C16	127.3 (16)	H19B—C19—H19C	109.5
C15—C11—C16	128.0 (17)	C15—C20—H20A	109.5
C11—C12—Ir1	70.3 (7)	C15—C20—H20B	109.5
C13—C12—Ir1	69.6 (7)	C15—C20—H20C	109.5
C17—C12—Ir1	125.5 (8)	H20A—C20—H20B	109.5
C11—C12—C13	109.8 (12)	H20A—C20—H20C	109.5
C11—C12—C17	124.1 (14)	H20B—C20—H20C	109.5
C13—C12—C17	126.2 (14)		
			
N2^i^—Ir1—N1—C2	106.2 (8)	C11—C12—C13—C14	−6.9 (13)
N2—Ir1—N1—C2	31.3 (8)	C17—C12—C13—C14	174.6 (12)
C15—Ir1—N1—C2	−71.6 (14)	Ir1—C12—C13—C14	−65.8 (8)
C13—Ir1—N1—C2	−134.7 (8)	C11—C12—C13—C18	−176.0 (12)
C11—Ir1—N1—C2	−63.8 (9)	C17—C12—C13—C18	5.4 (19)
C12—Ir1—N1—C2	−97.1 (8)	Ir1—C12—C13—C18	125.1 (12)
C14—Ir1—N1—C2	−146.2 (9)	C11—C12—C13—Ir1	58.9 (8)
N2^i^—Ir1—N1—C6	−83.1 (9)	C17—C12—C13—Ir1	−119.7 (12)
N2—Ir1—N1—C6	−158.0 (9)	N1—Ir1—C13—C12	77.0 (7)
C15—Ir1—N1—C6	99.1 (14)	N2^i^—Ir1—C13—C12	169.3 (6)
C13—Ir1—N1—C6	36.0 (9)	N2—Ir1—C13—C12	−24 (2)
C11—Ir1—N1—C6	106.9 (9)	C15—Ir1—C13—C12	−79.5 (8)
C12—Ir1—N1—C6	73.6 (9)	C11—Ir1—C13—C12	−36.1 (8)
C14—Ir1—N1—C6	24.5 (12)	C14—Ir1—C13—C12	−114.0 (11)
C2—S1—N2—Ir1^i^	−78.5 (6)	N1—Ir1—C13—C14	−169.0 (7)
C2—S1—N2—Ir1	43.6 (5)	N2^i^—Ir1—C13—C14	−76.8 (8)
N1—Ir1—N2—S1	−42.5 (4)	N2—Ir1—C13—C14	89.8 (17)
N2^i^—Ir1—N2—S1	−130.0 (6)	C15—Ir1—C13—C14	34.5 (8)
C15—Ir1—N2—S1	113.2 (6)	C11—Ir1—C13—C14	77.9 (9)
C13—Ir1—N2—S1	62.1 (17)	C12—Ir1—C13—C14	114.0 (11)
C11—Ir1—N2—S1	73.2 (6)	N1—Ir1—C13—C18	−42.9 (14)
C12—Ir1—N2—S1	43.1 (7)	N2^i^—Ir1—C13—C18	49.4 (15)
C14—Ir1—N2—S1	135.3 (6)	N2—Ir1—C13—C18	−144.1 (15)
N1—Ir1—N2—Ir1^i^	87.6 (4)	C15—Ir1—C13—C18	160.6 (15)
N2^i^—Ir1—N2—Ir1^i^	0.0	C11—Ir1—C13—C18	−156.0 (15)
C15—Ir1—N2—Ir1^i^	−116.8 (5)	C12—Ir1—C13—C18	−119.9 (16)
C13—Ir1—N2—Ir1^i^	−167.8 (15)	C14—Ir1—C13—C18	126.1 (16)
C11—Ir1—N2—Ir1^i^	−156.8 (5)	C12—C13—C14—C15	8.5 (14)
C12—Ir1—N2—Ir1^i^	173.1 (4)	C18—C13—C14—C15	177.1 (13)
C14—Ir1—N2—Ir1^i^	−94.7 (7)	Ir1—C13—C14—C15	−56.1 (10)
C6—N1—C2—C3	−1.4 (16)	C12—C13—C14—C19	−170.4 (13)
Ir1—N1—C2—C3	169.7 (8)	C18—C13—C14—C19	−2(2)
C6—N1—C2—S1	178.4 (8)	Ir1—C13—C14—C19	125.0 (13)
Ir1—N1—C2—S1	−10.5 (11)	C12—C13—C14—Ir1	64.7 (8)
N2—S1—C2—N1	−21.7 (9)	C18—C13—C14—Ir1	−126.8 (13)
N2—S1—C2—C3	158.2 (9)	N1—Ir1—C14—C15	139.6 (8)
N1—C2—C3—C4	−2.2 (18)	N2^i^—Ir1—C14—C15	−119.5 (8)
S1—C2—C3—C4	177.9 (9)	N2—Ir1—C14—C15	−36.7 (11)
C2—C3—C4—C5	5(2)	C13—Ir1—C14—C15	121.8 (11)
C3—C4—C5—C6	−5(2)	C11—Ir1—C14—C15	38.3 (8)
C4—C5—C6—N1	1.3 (19)	C12—Ir1—C14—C15	82.1 (9)
C2—N1—C6—C5	1.9 (16)	N1—Ir1—C14—C13	17.9 (11)
Ir1—N1—C6—C5	−168.7 (8)	N2^i^—Ir1—C14—C13	118.7 (7)
N1—Ir1—C11—C16	62.4 (18)	N2—Ir1—C14—C13	−158.4 (6)
N2^i^—Ir1—C11—C16	−92 (2)	C15—Ir1—C14—C13	−121.8 (11)
N2—Ir1—C11—C16	−18.7 (18)	C11—Ir1—C14—C13	−83.4 (8)
C15—Ir1—C11—C16	−123 (2)	C12—Ir1—C14—C13	−39.7 (7)
C13—Ir1—C11—C16	158.4 (18)	N1—Ir1—C14—C19	−97.1 (19)
C12—Ir1—C11—C16	123 (2)	N2^i^—Ir1—C14—C19	3.8 (19)
C14—Ir1—C11—C16	−158.7 (19)	N2—Ir1—C14—C19	86.6 (19)
N1—Ir1—C11—C12	−60.2 (8)	C15—Ir1—C14—C19	123 (2)
N2^i^—Ir1—C11—C12	145.6 (9)	C13—Ir1—C14—C19	−115 (2)
N2—Ir1—C11—C12	−141.3 (7)	C11—Ir1—C14—C19	162 (2)
C15—Ir1—C11—C12	114.8 (11)	C12—Ir1—C14—C19	−155 (2)
C13—Ir1—C11—C12	35.8 (7)	C13—C14—C15—C11	−7.1 (15)
C14—Ir1—C11—C12	78.7 (8)	C19—C14—C15—C11	171.7 (14)
N1—Ir1—C11—C15	−175.0 (7)	Ir1—C14—C15—C11	−63.0 (10)
N2^i^—Ir1—C11—C15	30.7 (15)	C13—C14—C15—C20	−177.7 (13)
N2—Ir1—C11—C15	103.8 (8)	C19—C14—C15—C20	1(2)
C13—Ir1—C11—C15	−79.1 (9)	Ir1—C14—C15—C20	126.4 (14)
C12—Ir1—C11—C15	−114.8 (11)	C13—C14—C15—Ir1	55.9 (9)
C14—Ir1—C11—C15	−36.1 (8)	C19—C14—C15—Ir1	−125.3 (15)
C16—C11—C12—C13	−179.2 (14)	C16—C11—C15—C14	−175.3 (15)
C15—C11—C12—C13	2.7 (13)	C12—C11—C15—C14	2.8 (15)
Ir1—C11—C12—C13	−58.5 (8)	Ir1—C11—C15—C14	65.0 (10)
C16—C11—C12—C17	−1(2)	C16—C11—C15—C20	−4(2)
C15—C11—C12—C17	−178.7 (11)	C12—C11—C15—C20	174.0 (12)
Ir1—C11—C12—C17	120.1 (11)	Ir1—C11—C15—C20	−123.8 (13)
C16—C11—C12—Ir1	−120.7 (15)	C16—C11—C15—Ir1	119.7 (16)
C15—C11—C12—Ir1	61.2 (9)	C12—C11—C15—Ir1	−62.2 (8)
N1—Ir1—C12—C13	−109.7 (7)	N1—Ir1—C15—C14	−107.4 (13)
N2^i^—Ir1—C12—C13	−24.3 (14)	N2^i^—Ir1—C15—C14	75.1 (9)
N2—Ir1—C12—C13	172.8 (6)	N2—Ir1—C15—C14	155.9 (8)
C15—Ir1—C12—C13	81.4 (8)	C13—Ir1—C15—C14	−36.0 (8)
C11—Ir1—C12—C13	121.1 (11)	C11—Ir1—C15—C14	−118.3 (12)
C14—Ir1—C12—C13	39.9 (8)	C12—Ir1—C15—C14	−79.0 (9)
N1—Ir1—C12—C11	129.2 (8)	N1—Ir1—C15—C11	10.9 (16)
N2^i^—Ir1—C12—C11	−145.4 (10)	N2^i^—Ir1—C15—C11	−166.5 (7)
N2—Ir1—C12—C11	51.7 (9)	N2—Ir1—C15—C11	−85.8 (8)
C15—Ir1—C12—C11	−39.7 (9)	C13—Ir1—C15—C11	82.3 (8)
C13—Ir1—C12—C11	−121.1 (11)	C12—Ir1—C15—C11	39.3 (8)
C14—Ir1—C12—C11	−81.2 (9)	C14—Ir1—C15—C11	118.3 (12)
N1—Ir1—C12—C17	10.8 (14)	N1—Ir1—C15—C20	127.5 (16)
N2^i^—Ir1—C12—C17	96.2 (15)	N2^i^—Ir1—C15—C20	−50.0 (19)
N2—Ir1—C12—C17	−66.7 (15)	N2—Ir1—C15—C20	30.8 (19)
C15—Ir1—C12—C17	−158.1 (15)	C13—Ir1—C15—C20	−161 (2)
C13—Ir1—C12—C17	120.5 (16)	C11—Ir1—C15—C20	117 (2)
C11—Ir1—C12—C17	−118.4 (16)	C12—Ir1—C15—C20	156 (2)
C14—Ir1—C12—C17	160.4 (15)	C14—Ir1—C15—C20	−125 (2)

Symmetry codes: (i) −*x*+1, −*y*, −*z*.

### Hydrogen-bond geometry (Å, °)

**Table:**

*D*—H···*A*	*D*—H	H···*A*	*D*···*A*	*D*—H···*A*
N2—H2···I1^i^	0.91	2.91	3.64 (1)	139

Symmetry codes: (i) −*x*+1, −*y*, −*z*.
